# A CNTFET based process variation resilient SRAM design for stable low power and half select free operation

**DOI:** 10.1038/s41598-026-48572-6

**Published:** 2026-04-22

**Authors:** Shams Ul Haq, Alireza Aminzadeh, Abdolreza Darabi, Erfan Abbasian, Owais Ahmad Shah, Vakkalakula Bharath Sreenivasulu

**Affiliations:** 1 Department of Electronics and Communication Engineering, Jamia Millia Islamia, New Delhi, 110025 India; 2 Department of Electrical and Computer Engineering, Tarbiat Modares University, Tehran, Iran; 3 Department of Electrical Engineering, Shiraz University of Technology (SUTech), Shiraz, 71557-13876 Iran; 4 Department of Electrical and Computer Engineering, Babol Noshirvani University of Technology, Babol, 47148-71167 Iran; 5 Department of Electronics and Communication Engineering, Dayananda Sagar University, Bengaluru, 562112 India; 6 Department of Electronics and Communication Engineering, Manipal Institute of Technology Bengaluru, Manipal Academy of Higher Education, Manipal, India

**Keywords:** SRAM, Carbon nanotube FETs, Low power, Monte-Carlo simulation, Single-ended structure

## Abstract

Static random-access memory (SRAM) design at nanoscale dimensions faces critical challenges arising from degraded stability, excessive power dissipation, and heightened sensitivity to process variations, particularly under low-voltage operation. To address these limitations, this paper proposes a robust and energy-efficient carbon nanotube field-effect transistor (CNTFET)-based nine-transistor (9T) SRAM cell architecture optimized for low-power applications. The proposed design employs a fully decoupled read and write structure with a single-ended access scheme, effectively eliminating read-disturb and half-select failures while enhancing overall noise immunity. Read stability is significantly improved by isolating the storage nodes from the read bitline, enabling the read static noise margin (RSNM) to reach the hold static noise margin (HSNM). Write robustness is achieved through controlled manipulation of the inverter pull-down paths, facilitating conflict-free write operations without aggressive transistor upsizing or complex assist circuitry. HSPICE simulations using the Stanford 32-nm CNTFET model demonstrate that, at a supply voltage of 0.3 V, the proposed SRAM achieves a 2.1 × improvement in RSNM and over a 14 × enhancement in write static noise margin (WSNM) compared to the conventional 6T SRAM. In addition, reduced bitline activity, elimination of precharge circuitry, and effective transistor stacking result in substantial reductions in read, write, and leakage power consumption. Monte Carlo simulations incorporating realistic process variations further confirm superior robustness, with the highest mean-to-standard-deviation ratios for both RSNM and WSNM among the compared designs. Layout-level evaluation shows that these benefits are achieved with only a modest area overhead relative to the 6T SRAM cell and with a smaller footprint than existing 9T and 10T alternatives. Overall, the proposed CNTFET-based 9T SRAM cell provides a well-balanced solution for low-voltage, energy-constrained, and variability-aware memory systems, making it a promising candidate for future CNTFET-based integrated circuits.

## Introduction

The proliferation of internet of things (IoT) applications has led to an exponential increase in the deployment of wireless sensor nodes, which form the foundational layer of pervasive sensing networks. These nodes, often operating under stringent energy budgets harvested from their environment, necessitate ultra-low-power system-on-chip (SoC) designs^1^. Embedded static random-access memories are indispensable components in these sensor interfaces, data processors, and communication modules, occupying a substantial portion of the chip area and dominating the overall power consumption and performance^2^. Nowadays, the trend of continuous and intensive scaling of technology nodes towards single digits has provided significant challenges in designing SRAM architectures in SoC platforms with a significant share of area for very-large-scale integration (VLSI) circuit designers^3^. These challenges include data-dependent leakage in the read path, write degradation, significant leakage currents, and limited stability, especially when operating under reduced supply voltage conditions, which has led to the reliability of conventional SRAMs with a simple six-transistor structure^4–7^. On the other hand, the robustness of SRAM cells based on silicon complementary metal–oxide–semiconductor (CMOS) technology has faced very important limitations such as reduced static noise margins (SNMs), increased leakage power, and extreme sensitivity to process changes, especially with reduced cell supply voltages and aggressive technology scaling^8,9^. Therefore, emerging CMOS-compatible technology along with memory architectures relying on effective transistor-level methods have become very important for designers to address and overcome the challenges of scalability^10,11^. From a technological perspective, emerging devices such as fin-shaped field effect transistors (FinFETs) and carbon nanotube-based field effect transistors have been introduced as alternatives to conventional silicon (Si)-based MOSFETs with strong electrostatic properties to improve the reliability performance of memory designs in low-voltage applications and have attracted considerable research attention^12,13^. As multi-gate devices, FinFETs, although improving electrostatic control, present non-trivial challenges related to fabrication complexity, threshold voltage variability, and thermal reliability^14,15^. In contrast, CNTFETs with their unique properties of near-ballistic carrier transport, excellent electrostatic control, high scalability, and fine-tuning of threshold voltage based on engineering selection of physical parameters of nanotubes have attracted wide attention of researchers as promising candidates in the design of integrated circuits^16–19^. Also, from the perspective of circuit-level methods in the design of memory structures with ensuring reliable performance at the nanoscale, we can mention dual-threshold techniques, read-decoupled with isolation of storage nodes from the read BL to significantly improve read-SNM (RSNM), and write-assisted techniques such as feedback cutoff, virtual ground, power gating, and transistor stacking to improve write-SNM (WSNM)^11–13,20–22^ So far, a wide range of SRAM architectures with different transistor topologies, latch-based techniques with different inverter structures in CNTFETs technology have been proposed by designers to overcome the scaling limitations of conventional technologies^13,18,23–25^.

Many proposed designs suffer from the inability to implement in bit-interleaved architectures due to increased circuit control complexity and area overhead, high trade-offs between safety parameters in terms of noise, dynamic power, and write delay, as well as remaining vulnerable to half-select disturbance. The consequence of these problems is that achieving an optimal balance between other key and challenging parameters of stability, power, delay, and area remains a challenge for the design of SRAM architectures. In this regard, a nine-transistor (9T) SRAM cell with a novel transistor-level architecture based on CNTFETs technology has been proposed, which, by combining circuit-level techniques, simultaneously addresses challenges such as read stability, write robustness, leakage power, and sensitivity to process variations at very low voltages. The proposed memory architecture has several unique features, which can be summarized as follows:

- Complete separation of read and write paths and better transistor scaling compatible with implementation in bit-interleaved structure and complete elimination of half-selection errors.
- Targeted control in the pull-down network of inverters in the write phase without changing the transistor size or complex auxiliary circuits and increasing the overhead.
- Significant reduction of hold-state leakage based on the use of effective stacking of transistors in the latch core and floating BL read.
- Significant reduction of dynamic read power based on the use of a single-ended read scheme without BL precharge.
- Ensuring stable operation at low cell voltages and increasing tolerance to process variations by using the specific threshold tuning capability of CNTFETs.

Extensive simulation results in the Synopsys HSPICE simulator based on the Stanford University 32 nm CNTFET model indicate that the SRAM cell with the proposed architecture has a RSNM has achieved very close to the hold-SNM at an operating supply voltage of 0.3 V, where it provides a 2.1 × improvement in RSNM over the conventional 6T cell structure. In addition, by weakening the contention in the write path through disconnection of the pull-down network within the proposed memory structure, more than 14 × improvement is achieved for the WSNM parameter. The encouraging results from the power analysis show that the proposed SRAM architecture has the lowest read, write, and leakage power among the other prior architectures, with leakage power being 56.1%, 42.9%, 25.0%, and 35.7% lower than the 6T, OSST10T, SEHSF9T, and SEFC10T designs, respectively. On the other hand, Monte Carlo (MC) simulations considering realistic process variations also confirm the high robustness of the proposed memory design, and it also has the highest mean to standard deviation ratio (μ/σ) for both RSNM and WSNM metrics. Additionally, the evaluation at the layout level confirms these improvements with only an area overhead of 10.3% compared to the conventional six-transistor SRAM cell, where the area occupied by the proposed design is less than most of the reported 9T and 10T designs. Finally, the proposed SRAM cell is considered a very good candidate for designing next-generation integrated architectures based on emerging technologies by providing a balanced, low-power, and robust solution to process variations.

Unlike existing 9T and 10T CNTFET SRAM designs that rely on assist circuitry or increased structural complexity, the proposed architecture introduces a transistor-level write facilitation mechanism based on selective pull-down network control using a VGND signal, combined with a compact decoupled read path. This enables simultaneous improvement in RSNM, WSNM, and power efficiency while maintaining low design complexity and area overhead.

Unlike existing read-decoupled and write-assisted SRAM designs that rely on external assist techniques such as power gating, wordline boosting, or feedback cutting, the proposed architecture introduces an intrinsic transistor-level write facilitation mechanism based on VGND-controlled pull-down modulation within the cross-coupled inverter pair. This enables dynamic weakening of the latch during write operations, ensuring conflict-free switching without additional assist circuitry. Furthermore, a compact decoupled read path is employed to fully isolate the storage nodes, achieving improved read stability without increasing design complexity.

The remaining sections of this paper are structured as follows. Section “CNTFET technology” describes the fundamental concepts of CNTFET technology along with their performance. Section “Prior CNTFET SRAM works” provides a comprehensive review and description of previous SRAM architectures based on CNTFET technology. The transistor level structure of the proposed 9T SRAM cell along with a detailed description of its performance trend is introduced in Section “Structure and operations of proposed 9T SRAM cell”. Section “Results and analysis” of this paper presents the simulation results, statistical analysis, and comparison of the proposed design with advanced peer designs. Finally, the conclusion of the paper is presented in Section “Conclusion”.

## CNTFET technology

CNTFETs have gained significant attention as promising candidates for nanoscale integrated circuits due to their superior electrostatic properties and excellent carrier transport capabilities. The operation and performance of CNTFETs are fundamentally determined by the structural and electronic characteristics of carbon nanotubes, which serve as the channel material in these devices.

Carbon nanotubes (CNTs) are quasi one-dimensional nanostructures formed from ${sp}^{2}$-bonded carbon atoms arranged in a cylindrical geometry^13,14^. Based on their structural composition, carbon nanotubes are broadly categorized into single-wall and multi-wall configurations. Single-wall CNTs consist of a single atomic layer and exhibit well-defined and controllable electronic behavior, making them highly suitable for transistor applications. In contrast, multi-wall CNTs comprise multiple concentric shells, which enhance mechanical stability but introduce complex electrical interactions due to coupling between adjacent layers^20^.

**Fig. 1:**
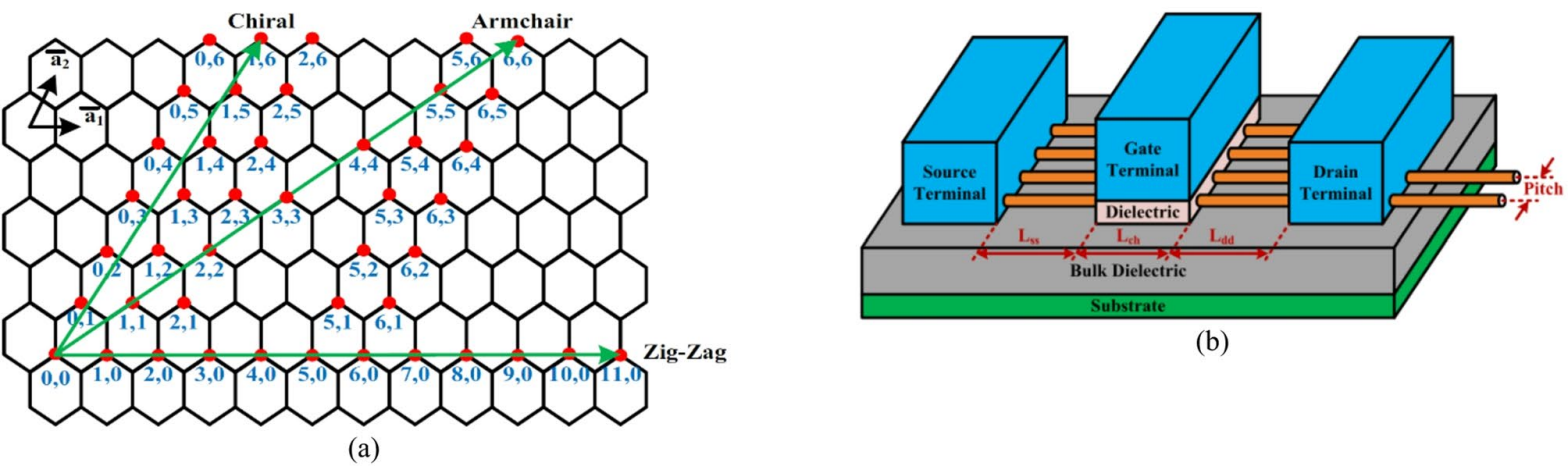
(**a**) Graphene sheet^7^ and (**b**) Physical structure of the CNTFET device^26^.

The electrical behavior of a CNT is governed by its atomic arrangement, which is defined by a pair of integers (*n*, *m*) describing the rolling direction of the graphene lattice, as illustrated in Fig. 1a^7^. This structural parameter determines whether a nanotube behaves as a metal or a semiconductor. The rolling configuration is mathematically represented by the chiral vector in Eq. (1), where the graphene lattice vectors ${\overrightarrow{a}}_{1}$ and ${\overrightarrow{a}}_{2}$ define the unit cell. CNTs exhibit metallic characteristics when the condition $m-n=3k$, where $k$ is an integer, is satisfied; otherwise, a finite bandgap exists, resulting in semiconducting behavior. This property enables selective utilization of nanotubes for electronic device fabrication^7^.

1 $$\overrightarrow {Chiral} = m\overrightarrow {{a_{1} }} + n\overrightarrow {{a_{2} }}$$

The physical diameter of a CNT is directly related to its chirality and can be calculated using Eq. (2). The nanotube diameter plays a critical role in determining key electronic parameters, including energy bandgap and carrier confinement^5^.

2 $$D_{CNT} = \frac{{a\sqrt {n^{2} + m^{2} + nm} }}{\pi }$$

In CNT-based transistors, the threshold voltage is closely linked to the bandgap energy of the nanotube channel and is inversely proportional to its diameter. This relationship is described by Eq. (3). As a result, the threshold voltage of CNTFETs can be precisely adjusted by selecting nanotubes with appropriate diameters and chirality indices. This inherent tunability provides a major advantage over conventional silicon-based devices^11^.

3 $$V_{th} \simeq \frac{{E_{g} }}{2e} = \frac{{aV_{\pi } }}{{\sqrt 3 eD_{CNT} }} = \frac{0.42}{{D_{CNT} }}$$

CNTFETs retain a MOSFET-like architecture, comprising gate, source, drain, and substrate terminals, while replacing the silicon channel with a semiconducting CNT. Both n-type and p-type CNTFETs can be realized, enabling complementary logic design. Depending on the contact engineering and doping strategy, CNTFETs are commonly classified into Schottky-barrier CNTFETs, band-to-band tunneling CNTFETs, and MOSFET-like CNTFETs. Among these, MOSFET-like CNTFETs offer higher on-state current and improved digital performance by eliminating Schottky barriers at the source and drain^7^. Consequently, MOSFET-like CNTFETs are adopted in this work, and their device structure is illustrated in Fig. 1b^26^. Table 1 shows the relationship between chirality, nanotube diameter, and threshold voltage for various CNT configurations.

**Table 1:** Carbon nanotube diameter and threshold voltage versus chirality vector in the CNTFET technology (calculated by Eqs. 2 and 3).

Chirality	${D}_{CNT}$(nm)	${V}_{th}$(V)
(8, 0)	0.781	0.559
(13, 0)	1.02	0.527
(14, 0)	1.114	0.392
(17, 0)	1.34	0.328
(19, 0)	1.487	0.289
(23, 0)	1.81	0.243
(25, 0)	1.956	0.223
(28, 0)	2.228	0.195

The effective gate width of a CNTFET is determined by the number of parallel nanotubes placed beneath the gate electrode and the spacing between adjacent nanotubes. This relationship is expressed in Eq. (4), where the gate width is defined as the greater of the minimum required width and the product of the number of nanotubes and their pitch. Accurate gate-width modeling is essential to ensure reliable operation and to optimize the performance and scalability of CNTFET-based integrated circuits^27^.

4 $$W_{Gate} = {\mathrm{max}}\left( {W_{min} ,N \times Pitch} \right)$$

## Prior CNTFET SRAM works

Among the emerging technologies in the continuous miniaturization of semiconductor technologies, CNTFET devices, as a potential candidate with the features highlighted in Section “CNTFET technology”, have been widely used in the design of efficient SRAM architectures to overcome the fundamental limitations of CMOS-based 6T cells at low cell supply voltages^13,27–29^.

One of the most widely used approaches to improve read reliability in SRAM architectures in CNTFET technology is the read path separation technique by electrically isolating the internal storage nodes from the read BL while receiving data from the cell. This transistor-level approach significantly improves the RSNM. On the other hand, since the write path in these structures does not change, it does not inherently improve the write stability significantly, leaving the WSNM largely unchanged^24,25,30^. To compensate for the write weakness, some designs proposed by researchers integrate the read isolation technique with write assistance mechanisms such as feedback cutting, floating storage nodes, or virtual ground/power supply^11,27,31,32^. These solutions effectively reduce the write contention and significantly improve the WSNM parameter, but in many cases they lead to increased write path length, increased dynamic power, or higher circuit layout and interconnection complexity, which can limit their application in systems with severe energy constraints^27,32^.

In another class of proposed designs, dual threshold voltage CNTFETs have been used to selectively enhance the pull-up or pull-down transistors, to significantly improve the read and write stability simultaneously. Despite achieving significant increases in the noise margins of the operational cycles, such structures are still prone to read disturbance problems due to incomplete isolation of the storage nodes during the data read phase^18^.

Some non-transistor SRAM architectures reduce read disturbance and limit switching activity and consequent dynamic power consumption by utilizing separate BLs for data read and write cycles. In these cell designs, transistor stacking is commonly used to reduce and suppress leakage current and improve stability in the data hold operation mode. However, considering the additional BL increases the complexity of the routing and transistor layout of the cell, and in many cases, the problem of half-select issue is not completely solved^24,25^.

Single-ended SRAM architectures with a separate read path topology by adding a dedicated access transistor further increase and improve the RSNM compared to the peer structures. However, although these structures provide a good improvement in read stability, they require the use of sense amplifiers due to the creation of unwanted and hidden current paths, which leads to increased design complexity and consequently energy overhead^11,28^. To improve robustness in the write phase, some ten-transistor SRAM structures use stacked p-type transistors and feedback cutoff techniques. Although these designs provide significant improvements in WSNM and achieve significant reductions in leakage power factor, the long write path in these designs increases write latency and limits performance in speed-critical applications^27,33^.

Another group of CNTFET SRAM cell designs utilizes power gating, in which the cell supply voltage is switched off during the write operation to simultaneously improve both RSNM and WSNM parameters. Despite the increased noise margins in the cells, this approach introduces limitations such as area overhead and additional leakage paths in the read BL compared to a conventional 6T cell^21,23^.

Ultimately, in^34^, the authors have developed compact seven-transistor SRAM cells based on CNTFETs by using the strategy of adding an always-on p-type transistor in the pull-up network to reduce leakage. However, these cells still inherit many of the major shortcomings of conventional six-transistor architectures, including sensitivity to half-selectivity disorder, limited noise margins, and high dynamic power. Overall, although SRAM designs based on emerging CNTFET technology have shown significant improvements in stability and power efficiency, achieving an optimal balance between other important parameters in SRAM structures, such as noise margins, power consumption, latency, and layout area, remains a major challenge. Therefore, future SRAM architectures must meet the needs of next-generation high-performance, low-power application platforms by intelligently combining various auxiliary techniques while minimizing circuit and physical overhead.

## Structure and operations of proposed 9 T SRAM cell

**Fig. 2:**
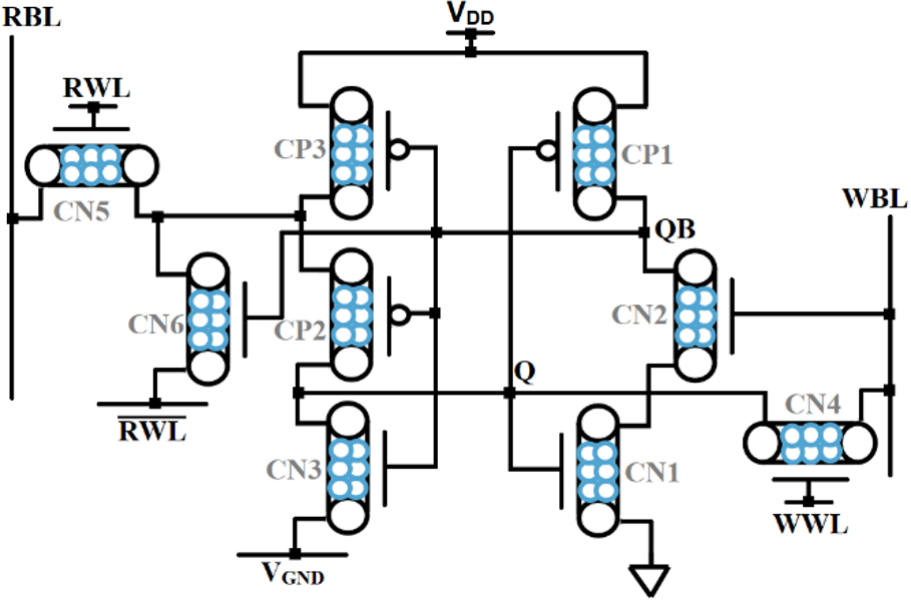
Structure of the proposed 9T SRAM cell.

Figure 2 and Table 2 respectively show the transistor level schematic of the proposed SRAM structure with nine transistors and the operational states of the control signals. According to the figure, it can be seen that the cell storage core consists of two hot-junction inverters that hold the data between themselves as a latch section. The first inverter consists of transistors CN1, CN2 and CP1, while the second inverter consists of transistors CN3, CP2 and CP3. The output of these two inverters creates complementary data sense nodes Q and QB that determine the logical state of the data in the cell. The write operation is controlled by transistor CN4, which is controlled by the write word line (WWL). When enabled, the write bit line (WBL) is directly connected to the storage node Q through transistor CN4. In order to facilitate the process of writing logical ‘1’ data, the source of transistor CN3 is connected to the virtual ground signal (VGND) which provides a controlled pull-down path for node Q. In contrast, applying a logical ‘0’ value is possible by connecting the gate of transistor CN2 to the control signal WBL. This mechanism reduces internal contention by selectively weakening the pull-down path of node QB and makes the writing process more stable. On the other hand, the read path is completely separated and managed by transistor CN5 which is controlled by the read word line (RWL). According to the figure, it can be seen that the read bit line (RBL) is responsible for extracting data from the latch section in the proposed memory structure. Finally, in the proposed structure, the source of transistor CN6 is connected to the complement of RWL, which isolates the read current path from the storage nodes and thus prevents the occurrence of read disturbances.

**Table 2:** Proposed 9T SRAM cell’s control signal status.

Signals	Hold	Read	Write ‘0’	Write ‘1’
RBL	Floating	Floating	Floating	Floating
RWL	GND	V_DD_	GND	GND
WBL	V_DD_	V_DD_	GND	V_DD_
WWL	GND	GND	V_DD_	V_DD_
VGND	GND	GND	GND	V_DD_

**Fig. 3:**
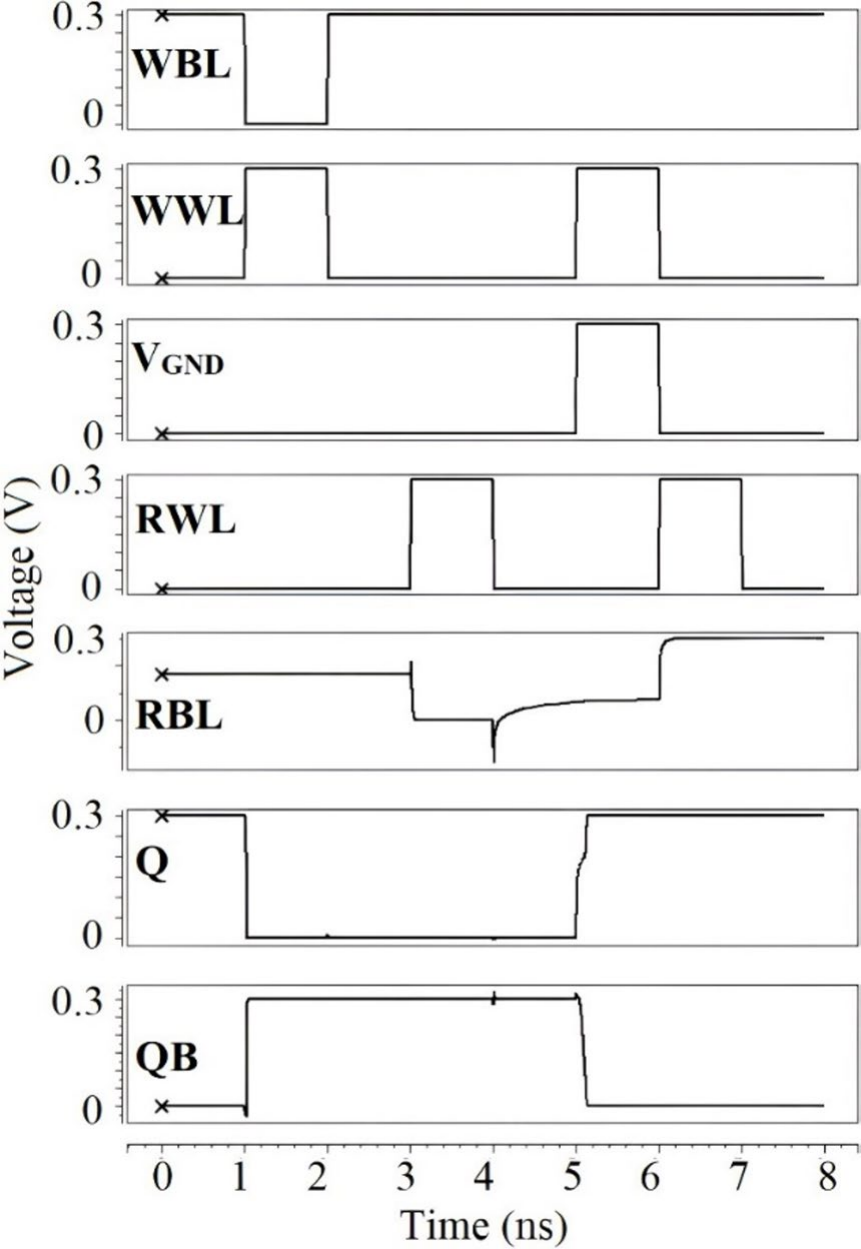
Timing diagram of the proposed 9T SRAM cell, showing sequence operations.

Figure 3 shows the timing diagram of the proposed memory cell. In the initial period of the diagram (0 to 1 ns), the cell is in the hold phase and the logical data value ‘1’ is stored in the Q node (and the logical value ‘0’ in the complementary QB node). To perform the data write cycle operation ‘0’, the WWL control signal is applied to the VDD level and the WBL is driven to ground (1 to 2 ns). After the write process is completed, the WWL control signal is deactivated and the WBL is returned to VDD again (time interval 2 to 3 ns). Next, the new data read operation is performed and by activating the RWL, the RBL is discharged and discharged to ground (3 to 4 ns). After the read is completed, the RWL returns to the zero voltage level (4 to 5 ns). Then, writing a logical value of ‘1’ is done by simultaneously applying the control signals WWL and VGND to the cell supply voltage level (5 to 6 ns). In order to check the accuracy of the stored data, RWL is re-activated and at this stage RBL is charged to the voltage level VDD (6 to 7 ns). Finally, the memory cell is placed in the data holding state by its latch section in the final interval of 7 to 8 ns.

### Hold mode

**Fig. 4:**
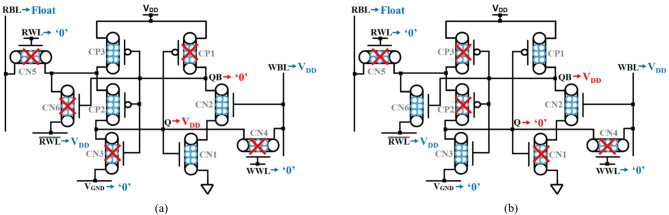
Hold-mode status of the proposed 9T SRAM cell. (**a**) hold ‘1’ and (**b**) hold ‘0’.

During the hold or standby state, the memory cell is inactive and does not perform read or write accesses; its sole function is to retain its existing data value without disturbance. To achieve this in the proposed 9T SRAM cell, specific control signals are applied. The WBL is connected to the V_DD_ and the RBL is left floating. Simultaneously, both the RWL and the WWL are forced to ground potential. This configuration has a direct effect on the cell’s internal access paths. Applying ground to RWL deactivates the transistor forming the read access path (CN5), while grounding WWL turns off the transistor of the write access path (CN4). By isolating the core storage nodes (Q and QB) from the external bitlines, the cross-coupled inverter latch is free to reinforce and maintain the stored logic state through positive feedback without any external interference. The specific voltage conditions and conductive states of all transistors for the two possible stored values are illustrated in Fig. 4, with part (a) depicting the cell in hold ‘1’ mode and part (b) depicting the hold ‘0’ mode.

### Read operation

The proposed 9T SRAM cell eliminates the standard precharge requirement by driving the RBL to full-rail logic levels during a read access, with the final state resolved by a sense amplifier. A read operation is initiated by asserting the RWL. Data transfer to the BL occurs through one of two distinct conductive paths controlled by the cell’s internal state.

**Fig. 5:**
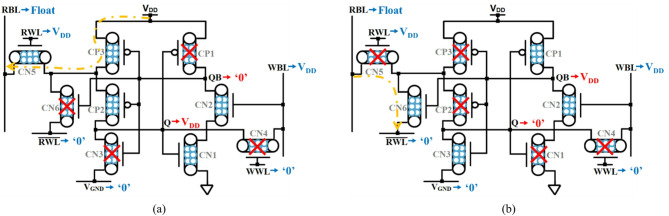
Read-mode status of the proposed 9T SRAM cell. (**a**) read ‘1’ status and (**b**) read ‘0’ status.

For a cell storing a logic ‘1’ at node Q (and ‘0’ at QB), the low QB voltage enables the p-type pull-up network CP2-CP3. This action charges the RBL via the CP3-CN5 path. The series n-type transistor CN5 causes a threshold voltage drop, resulting in a high RBL level of ${V}_{DD} - {V}_{th-CN5}$. This degraded but detectable logic high can be strengthened using a performance-enhancing keeper circuit, such as the positive-feedback design from reference^35^. Moreover, using a chirality of (28, 0) assigned to CN5, it is possible to reduce this voltage drop significantly. The specific waveform for this read ‘1’ operation is depicted in Fig. 5a.

When the cell stores a logic ‘0’ at node Q, transistor CN5 is activated. This creates a discharge path (CN5-CN6) that pulls the RBL to a solid logic low. The corresponding signal behavior for reading a ‘0’ is shown in Fig. 5b. This dual-mode read mechanism, illustrated in the figures, allows the proposed SRAM design to function correctly without a precharge phase.

Although the read ‘1’ operation results in a degraded high level due to the threshold voltage drop across the access transistor, reliable sensing is ensured using a single-ended sense amplifier with a skewed switching threshold. The significant voltage difference between the discharged (‘0’) and partially charged (‘1’) RBL enables robust detection. Furthermore, device-level optimization and optional keeper circuits can be employed to further improve the sensing margin.

### Write operation

To configure the memory cell for a write cycle, the RBL is left floating while the RWL is maintained at ground potential. The actual write process is triggered by asserting the WWL to a high logic level and presenting the intended data on the WBL. The logic level present on the WBL directly dictates the final state of the primary storage node Q: applying a ‘0’ writes a ‘0’, and applying a ‘1’ writes a ‘1’.

**Fig. 6:**
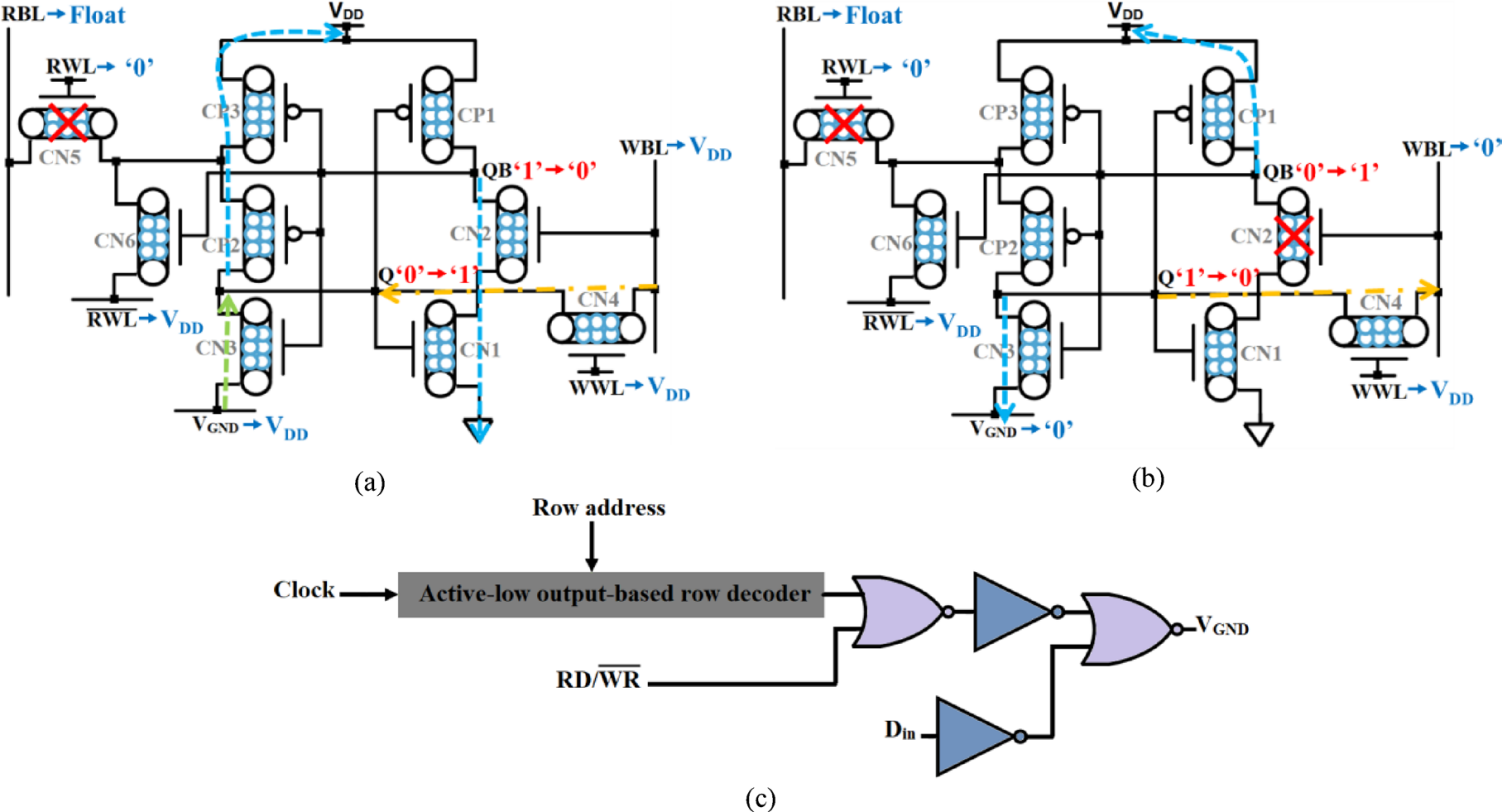
Write-mode status of the proposed 9T SRAM cell. (**a**) write ‘1’, (**b**) write ‘0’, (**c**) V_GND_ circuit generation in the proposed 9T SRAM cell.

For the case of writing a logic ‘1’ to a node Q that initially stores a ‘0’, the WBL is set to V_DD_. In this state, the source terminal of the transistor CN6 connected with the V_GND_ (= ‘1’) injects a logic ‘1’ to Q node in the initial time of writing process. This eliminates any active pull-down force on the node holding the existing ‘0’. This allows node Q to be pulled high without contention, charging to V_DD_ through transistor CN4. Once the voltage at Q exceeds the input trip point of the cross-coupled inverter pair, the n-channel transistor CN1 in the right inverter activates, driving the complementary node QB to ground. This operational sequence is detailed in Fig. 6a.

Conversely, to overwrite a logic ‘1’ at node Q with a ‘0’, the WBL is driven to ground and V_GND_ is set to logic ‘0’. This action deactivates transistor CN2, effectively weakening the right inverter and diminishing its capability to hold QB low. With CN2 off, node Q discharges to ground via the path through transistor CN5. The subsequent drop in Q’s voltage enables transistor CP1 in the right inverter to turn on, which then charges node QB to V_DD_. The complete process for writing a ‘0’ is captured in Fig. 6b. This dual-path write mechanism enables reliable state transitions within the 9T SRAM cell by selectively controlling the strengths of the cross-coupled inverter feedback loops.

Figure 6c depicts the circuit for generating the V_GND_ signal. Its operation proceeds as follows. During a write cycle, the active-low row decoder output goes to logic ‘0’ while the RD/$\overline{\mathrm{WR} }$ enable signal is pulled to ground. This causes the first NOR gate to output a ‘1’, which is then inverted to a ‘0’ before reaching the second NOR gate. Writing a ‘1’ occurs when the input data line D_in_ is raised to V_DD_, producing a ‘1’ at the V_GND_ output; writing a ‘0’ happens when D_in_ is low, resulting in a ‘0’ at V_GND_. For both read and hold modes, the V_GND_ output is fixed at ‘0’. During a read, the RD/$\overline{\mathrm{WR} }$ signal is high, forcing the first NOR gate to ‘0’; after inversion, this provides a constant ‘1’ to the second NOR gate, which locks V_GND_ at ‘0’. Similarly, in hold mode, the row decoder output is ‘1’, which also drives the first NOR gate to ‘0’ and consequently disables the V_GND_ output, holding it at ‘0’.

## Results and analysis

This section analyses the various performance metrics of the proposed 9T SRAM cell and compares them with the prior SRAM designs including 6T^36^, single-ended half-select-free 9T (SEHSF9T)^28^, one-sided schmitt-trigger 10T (OSST10T)^37^, and single-ended feedback-cutting 10T (SEFC10T)^27^ SRAM designs. The DC performance metrics (HSNM, RSNM, and WSNM) and transient performance metrics (read delay, write delay, read power, write power, and leakage power) of all the investigated SRAM designs were obtained using the Synopsis HSPICE simulator and the Stanford University’s CNTFET technology model files^16,17^. The most important parameters of the CNTFET technology are listed in Table 3.

**Table 3:** Various parameters of CNTFET technology.

Parameters	Values
Gate length	32 nm
Source/Drain length	32 nm
Gate oxide dielectric thickness	1.5 nm
CNT’s innertube space	6 nm
Top gate’s dielectric material constant	25
Fermi level	0.6 eV
Chirality vector	(19, 0)
Threshold voltage	0.289 V

To obtain accurate and practical simulation data, all SRAM cells were evaluated in an array arrangement. A 64 × 32 array (2 Kb) was implemented with an interconnect wire capacitance model of 0.16 fF per micrometer to capture timing and energy characteristics^38,39^. All reported performance measures are based on the central cell of this array. This cell provides the most stringent operating scenario, as it encounters the greatest wordline and bitline parasitic delays and experiences coupling interference from adjacent cells in both dimensions.

### Hold static noise margin

The HSNM quantifies the intrinsic DC noise tolerance of an SRAM cell in idle state. It is geometrically determined as the length of a side of the largest square that can be inscribed within the smaller lobe of the butterfly curve^20^. This curve is plotted from the VTCs of the cell’s cross-coupled inverters when the access transistors are disabled (hold mode). This analysis is essential for evaluating the fundamental stability of the storage latch, isolated from external access circuitry influences. A larger HSNM indicates greater robustness against static voltage disturbances and parameter variations during data retention^7^.

**Fig. 7:**
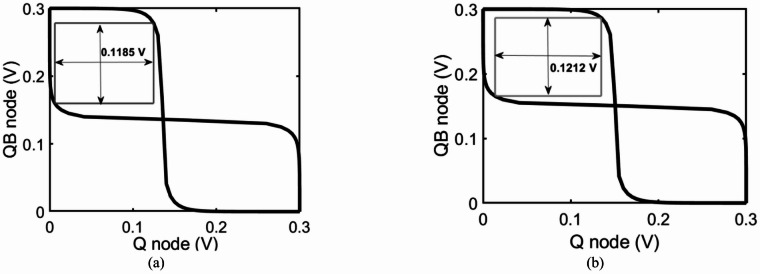
Butterfly curve obtained for (**a**) 6T and (**b**) proposed 9T in hold mode for measuring the HSNM value at 0.3 V supply voltage.

Figure 7 presents the hold-mode butterfly curves for the conventional 6T and the proposed 9T SRAM designs at a 0.3 V supply voltage, which are used to extract the HSNM. As shown, the proposed 9T exhibits an HSNM of 0.1212 V. This value represents a 1.023-fold improvement over the HSNM of the 6T. The enhancement is primarily due to the implementation of stacked transistors within the cross-coupled inverter pair, which increases the effective switching threshold voltage of the cell.

**Fig. 8:**
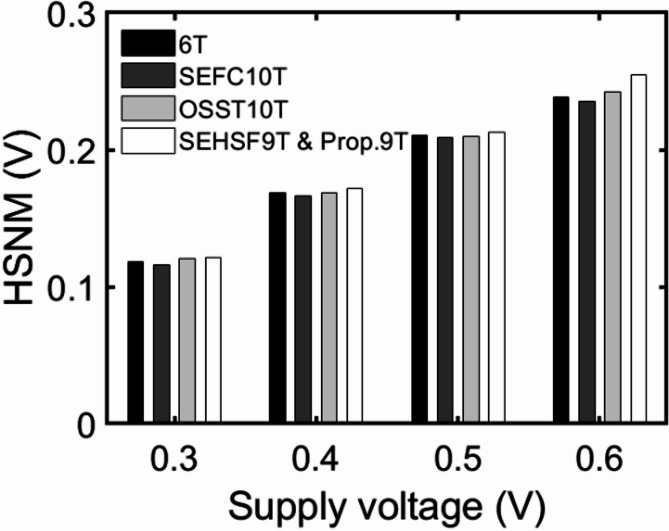
HSNM values of the investigated SRAM designs at a range of supply voltage values (0.3 to 0.6 V).

The HSNM values for all evaluated SRAM designs at a 0.3 V supply voltage are summarized in Table 4. The SEFC10T SRAM exhibits a lower HSNM than the conventional 6T SRAM. In contrast, the OSST10T SRAM achieves a higher HSNM due to its use of a Schmitt-trigger inverter combined with a standard inverter. The maximum HSNM is attained by the proposed and the SEHSF9T SRAM designs, which is a direct result of implementing stacked transistors in the pull-down network of their cross-coupled inverters. Figure 8 presents a comparison of the HSNM values for the investigated SRAM circuits across supply voltages from 0.3 to 0.6 V. The proposed design maintains a higher HSNM across the entire voltage range.

**Table 4:** HSNM values of the investigated SRAM designs ata a 0.3 V supply voltage value.

SRAM designs	HSNM (V)
6T^36^	0.1185
SEHSF9T^28^	0.1212
SEFC10T^27^	0.1159
OSST10T^37^	0.1204
Prop.9T	0.1212

### Read static noise margin

The RSNM is a critical metric quantifying SRAM cell stability during active read access. It is defined as the side length of the maximum square that can be inscribed within the smaller lobe of the butterfly curve generated under read-bias conditions^32^. The RSNM directly measures the cell’s vulnerability to the read-disturb effect, wherein a conduction path through the access transistor degrades the storage node voltage, potentially causing a destructive read operation. A sufficient RSNM is fundamental for ensuring read reliability^40^.

**Fig. 9:**
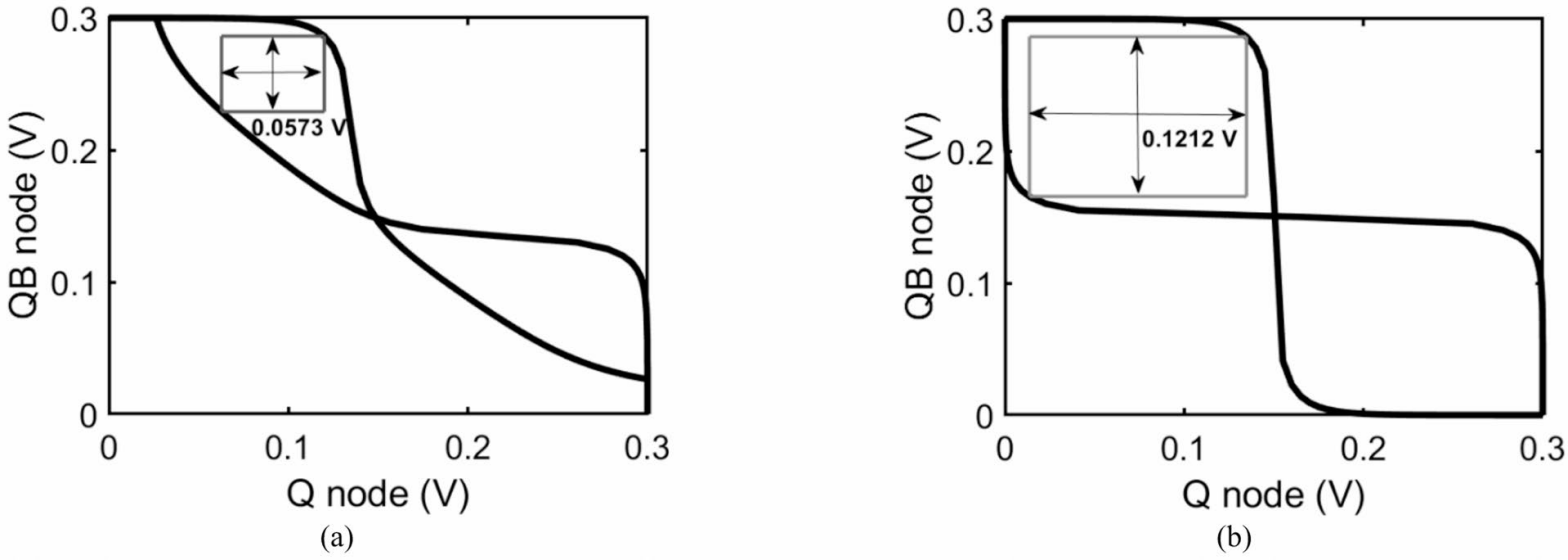
Butterfly curve obtained for (**a**) 6T and (**b**) proposed 9T in read mode for measuring the RSNM value at 0.3 V supply voltage.

**Fig. 10:**
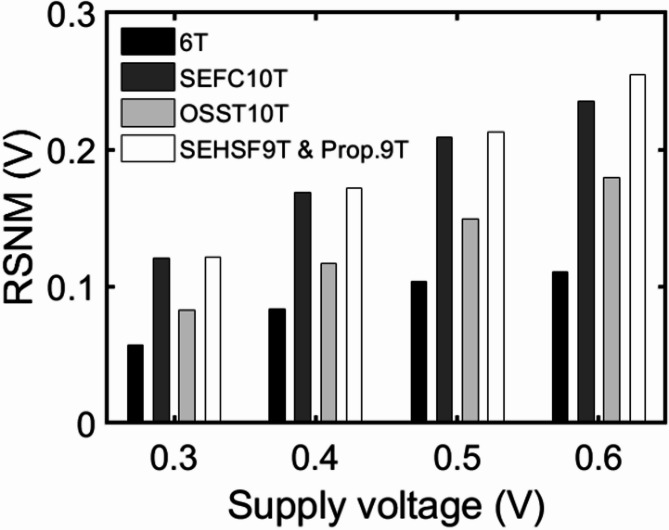
RSNM values of the investigated SRAM designs at a range of supply voltage values (0.3 to 0.6 V).

Figure 9 shows the butterfly curves in the read mode for extracting the RSNM parameter for two conventional and proposed six-transistor memory cells at a cell supply voltage of 0.3 V. According to the obtained curves, the proposed cell has achieved an RSNM value of 0.1212 V, which shows an improvement of 2.115 times compared to the conventional six-transistor reference cell. This increase in stability in the read phase is a consequence of the structural separation of the latch section cross-inverters from the BLs during the read cycle, and is an approach that does not completely eliminate the read disturbance mechanism present in the 6T architecture. Other RSNM values for the cells under study at a cell supply voltage of 0.3 V are presented in Table 5. Among the memory topologies studied, the architecture with the lowest RSNM value belongs to the 6T architecture, which is attributed to the read disturbance caused by the read/write path based on the common access transistor. On the other hand, the OSST10T architecture offers a higher noise margin due to the use of latch section inverters based on the Schmitt-trigger topology. Also, the proposed cells and the two memory architectures SEHSF9T and SEFC10T exhibit RSNM values equal to the HSNM resulting from the complete isolation of the storage nodes during the read operation. Among the other structures studied, the two proposed designs and SEHSF9T have the highest RSNM value by utilizing the stacking of transistors in the pull down of the inverters. The results of the comparison of the RSNM parameter in the supply voltage range of 0.3 to 0.6 V are shown in Fig. 10, which indicates that the proposed memory structure maintains superior read stability over the entire operating range.

**Table 5:** RSNM values of the investigated SRAM designs at a 0.3 V supply voltage value.

SRAM designs	RSNM (V)
6T^36^	0.0573
SEHSF9T^28^	0.1212
SEFC10T^27^	0.1204
OSST10T^37^	0.0829
Prop.9T	0.1212

### Write static noise margin

The WSNM parameter is considered a key indicator for measuring the robustness of write operations in SRAM architectures, and this parameter is extracted in the present study based on the composite word line margin (CWLM) criterion. In this method, the write capability of the cell is evaluated by gradually increasing the word line voltage from the logic level ‘0’ to the logic level ‘1’ for n-type access transistors. The critical switching threshold point in this process represents the minimum voltage required to create metastable instability (a quasi-stable transition) in the internal nodes of the cell^41^. In the framework of CWLM analysis, the value of the WSNM parameter is defined as the lowest word line voltage that is capable of inducing a state change in the storage latch architecture, where this value is determined from the intersection of the voltage transfer characteristics (VTC) of the cross-inverters under write-bias conditions. The main advantage of this approach is that it comprehensively models the electrical competition between the active access transistor and the storage feedback network, ultimately providing an accurate measure of the static writability^28^.

**Fig. 11:**
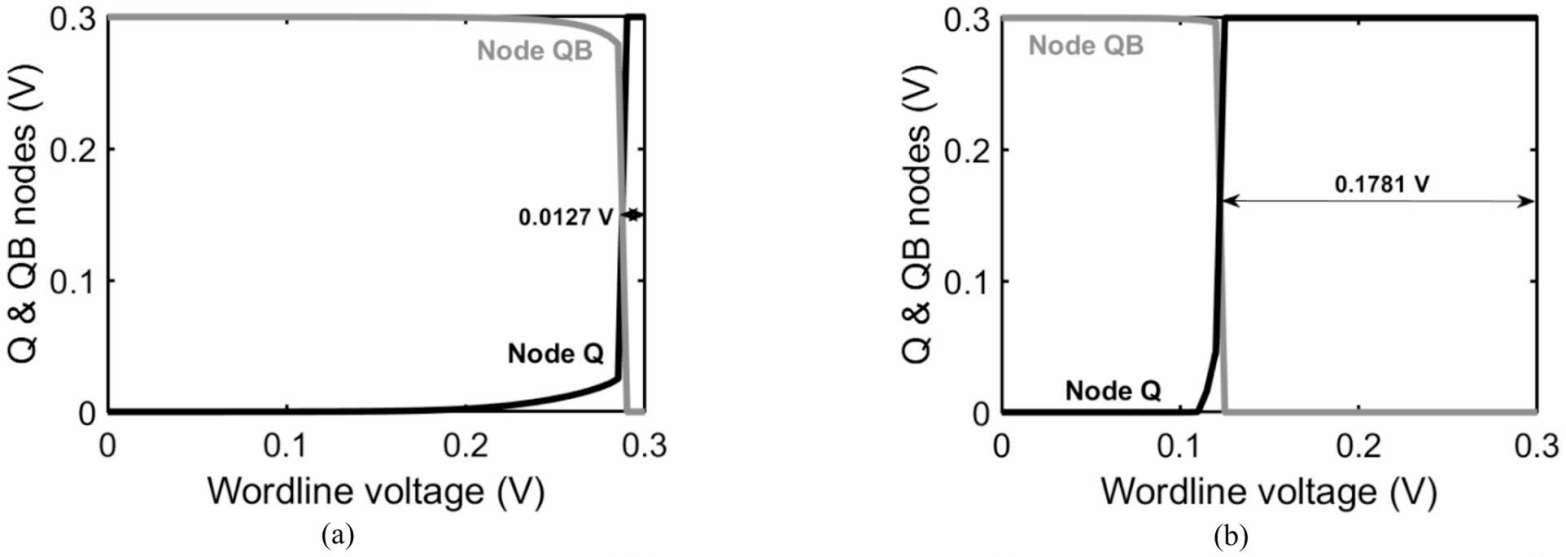
CWLM technique for (**a**) 6T and (**b**) proposed 9T in write mode for measuring the WSNM value at 0.3 V supply voltage.

Figure 11 shows the application of the CWLM method in extracting the WSNM factor for other conventional SRAM architectures and the proposed structure at a cell supply voltage of 0.3 V. This graph shows the voltage variations of the internal storage nodes in terms of the write word line voltage. According to the results, the proposed cell achieves a WSNM value of 0.1781 V, a significant improvement of 14.024 times compared to the 6 T cell. The significant increase in the write noise margin in the proposed memory architecture is mainly due to the use of a write assist mechanism, whereby the pull-down network of the node storing the logical value ‘0’ is conditionally disconnected during the write ‘1’ operation. This intervention weakens internal competition against the writing path, facilitates the process of changing the situation, and ultimately results in an increase in the writing margin.

**Fig. 12:**
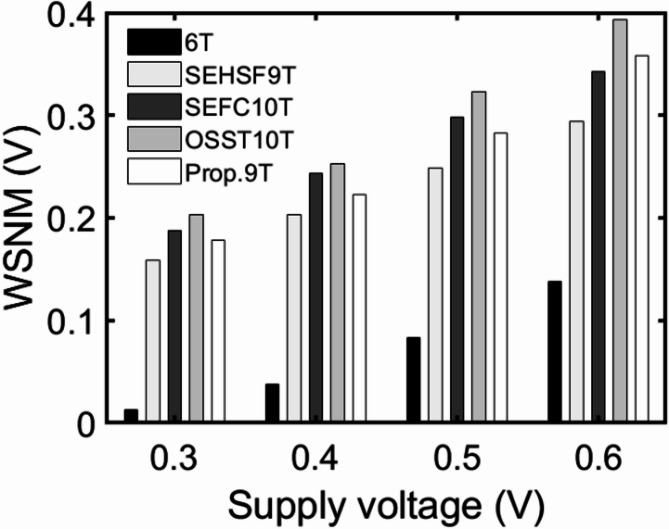
WSNM values of the investigated SRAM designs at a range of supply voltage values (0.3 to 0.6 V).

The WSNM for all investigated SRAM cells at a 0.3 V supply is summarized in Table 6. The conventional 6T exhibits the lowest WSNM, resulting from the absence of write-assist circuitry and inherent read/write access contention. In comparison, the SEHSF9T achieves a higher margin by employing a floating-node technique to write a logic ‘1’. The OSST10T attains the highest WSNM due to its pseudo dual-ended write structure integrated with a power-gating mechanism. The proposed 9T and SEFC10T designs demonstrate comparable WSNM performance. Figure 12 presents a comparison of the WSNM across a supply voltage range from 0.3 to 0.6 V.

**Table 6:** WSNM values of the investigated SRAM designs at a 0.3 V supply voltage value.

SRAM designs	WSNM (V)
6T^36^	0.0127
SEHSF9T^28^	0.1585
SEFC10T^27^	0.1878
OSST10T^37^	0.2031
Prop.9T	0.1781

### Read delay

The read speed of an SRAM cell is evaluated using its read access delay. To measure this delay, the signal controlling the read operation is activated in the SRAM designs under test. The measurement start time is defined as the instant when this control signal reaches 50% of the supply voltage (i.e. V_DD_). The measurement end time is defined as the moment when the corresponding bitline charges to $0.2\times {\mathrm{V}}_{\mathrm{DD}}$ or discharges to $0.8\times {\mathrm{V}}_{\mathrm{DD}}$ during the read operation^42^.

**Fig. 13:**
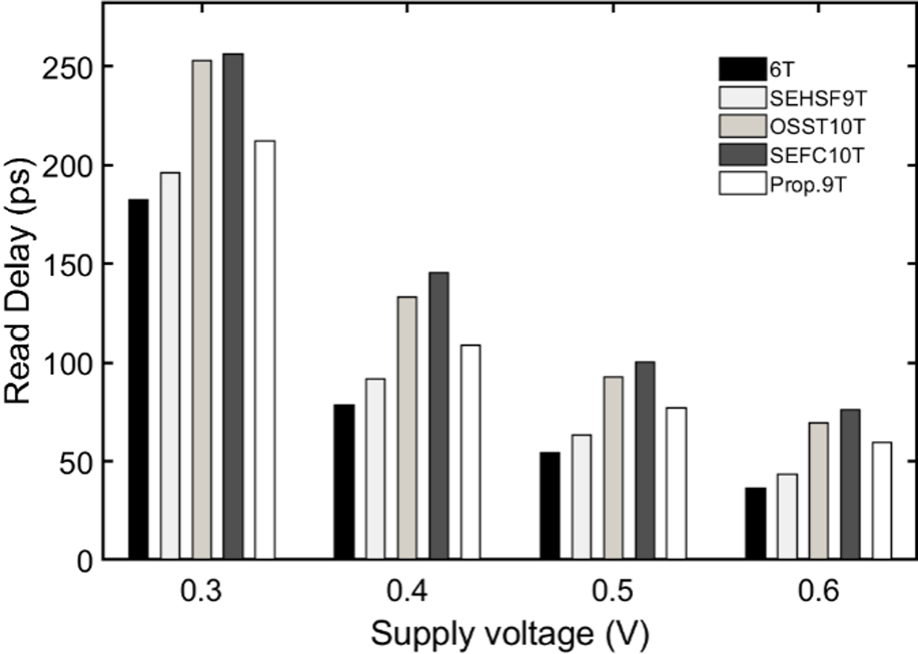
Read delay values of the investigated SRAM designs at a range of supply voltage values (0.3 to 0.6 V).

Figure 13 presents the measured read delay for the tested SRAM circuits across different supply voltages. As observed, the conventional 6T SRAM employs larger access transistors (to meet read‑write stability requirements), which increases the read current and consequently improves read speed. This design achieves the lowest read delay because it utilizes a fully differential sensing structure. In comparison, the SEHSF9T design exhibits a lower read delay than the proposed 9T SRAM design, as it employs only a single read access transistor.

When compared to the SEFC10T and OSST10T designs, the proposed circuit demonstrates higher read speed. This is because the read path in the OSST10T consists of three series transistors, while the SEFC10T read path contains two series transistors plus an additional control signal. This control signal must switch to a logic low during a read operation, which degrades the read speed.

At a supply voltage of 0.3 V, the proposed design increases the read delay by factors of 1.16 × and 1.08 × compared to the 6T and SEHSF9T designs, respectively. In contrast, when compared to the OSST10T and SEFC10T designs, it reduces the read delay by 16.11% and 17.25%, respectively.

### Write delay

The write speed of an SRAM, corresponding to the overwriting of stored data, is evaluated using the write access delay. To measure the write delay in the SRAM circuits under comparison, the control signal initiating the write operation is activated. The measurement start time is defined as the moment this control signal reaches 50% of the ${\mathrm{V}}_{\mathrm{DD}}$. The measurement end time is determined when the storage node Q reaches $0.9\times {\mathrm{V}}_{\mathrm{DD}}$ during a logic ‘1’ write operation^20^. Since the proposed SRAM employs a single-ended write structure, writing a logic ‘1’ is particularly challenging; therefore, the write- ‘1’ delay is the focus of this analysis.

**Fig. 14:**
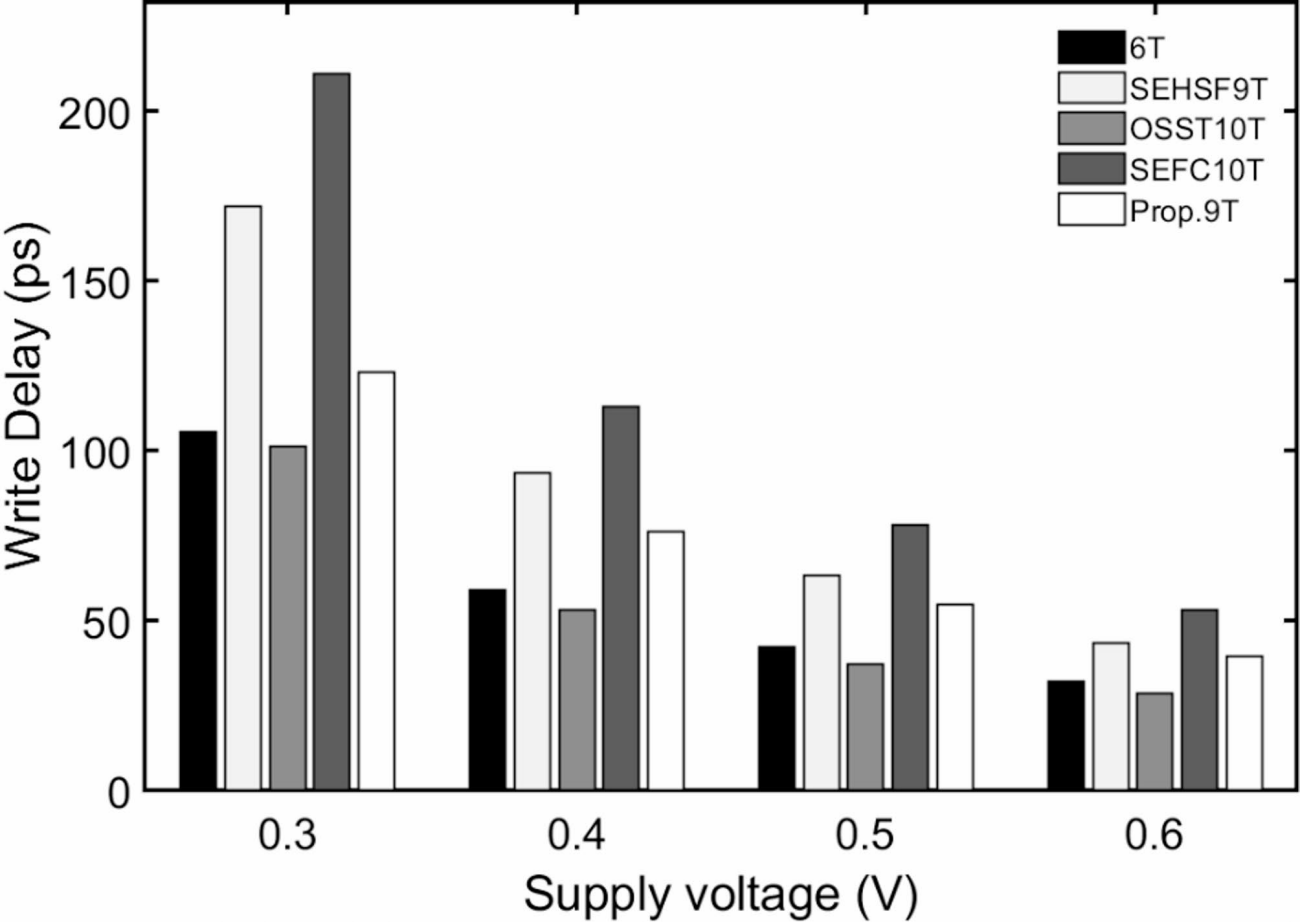
Write delay values of the investigated SRAM designs at a range of supply voltage values (0.3 to 0.6 V).

Figure 14 presents the measured write delays for the compared SRAM designs across various supply voltages. Among the designs, the conventional 6T SRAM utilizes a fully differential write structure, which enhances write speed. However, when compared to the OSST10T, it exhibits a larger write delay because the OSST10T employs a pseudo-differential write structure combined with a write-assist technique (power-gating technique).

Conversely, the proposed 9T SRAM design demonstrates a lower write delay compared to the SEHSF9T and SEFC10T designs. This is because the SEFC10T utilizes a write-assist technique involving feedback path cutting, which elongates the effective write path and increases the write delay. In other side, the SEHSF9T leverages a floating method to write a ‘1’, which is not an effect write-assist technique compared to the one used in the proposed design.

At a supply voltage of 0.3 V, the proposed 9T design exhibits a write delay of 123.28 ps. This represents a 16.9% increase compared to the 6T and a 28.3% increase compared to the OSST10T. However, the proposed design achieves a significant write speed improvement over the SEHSF9T and SEFC10T designs, reducing write delay by 28.3% and 41.6%, respectively.

### Dynamic power

The dynamic power consumed by an SRAM circuit for a read or write operation depends on its read/write structure (single-ended or differential), the charging/discharging of bitlines, and the switching of wordlines. SRAM designs with differential structures typically consume more power compared to single-ended designs, primarily due to the higher switching activity factor of their complementary bitlines. Furthermore, there is a direct relationship between the read/write speed of an SRAM cell and its dynamic power consumption; higher speed generally requires greater power^7,41^.

**Fig. 15:**
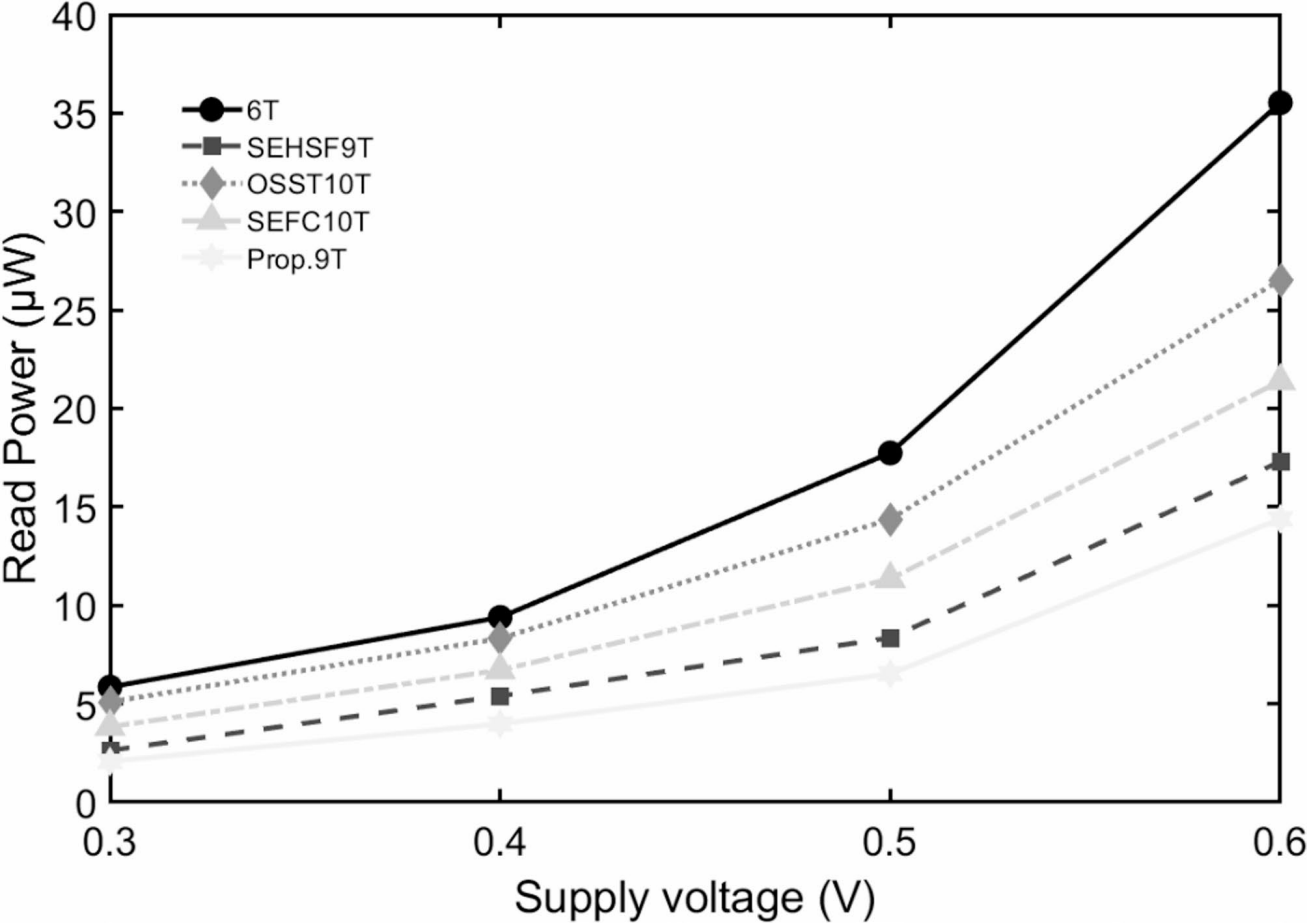
Read power values of the investigated SRAM designs at a range of supply voltage values (0.3 to 0.6 V).

Figure 15 shows the read power consumption for all compared SRAM circuits across various supply voltages. Among the designs, only the conventional 6T SRAM employs a fully differential reading structure, resulting in its higher read power consumption. In contrast, the proposed SRAM design achieves the lowest read power consumption. This is attributed to its elimination of the need for a bitline precharge circuit, leading to a significant reduction in read power. At a supply voltage of 0.3 V, the proposed 9T design achieves the lowest read power consumption of 2.07 µW. Compared to the other designs, it consumes 64.6% less read power than the 6T and 59.4% less than the OSST10T. It also offers moderate improvements over the SEHSF9T and SEFC10T, reducing read power by 21.3% and 46.0%, respectively.

**Fig. 16:**
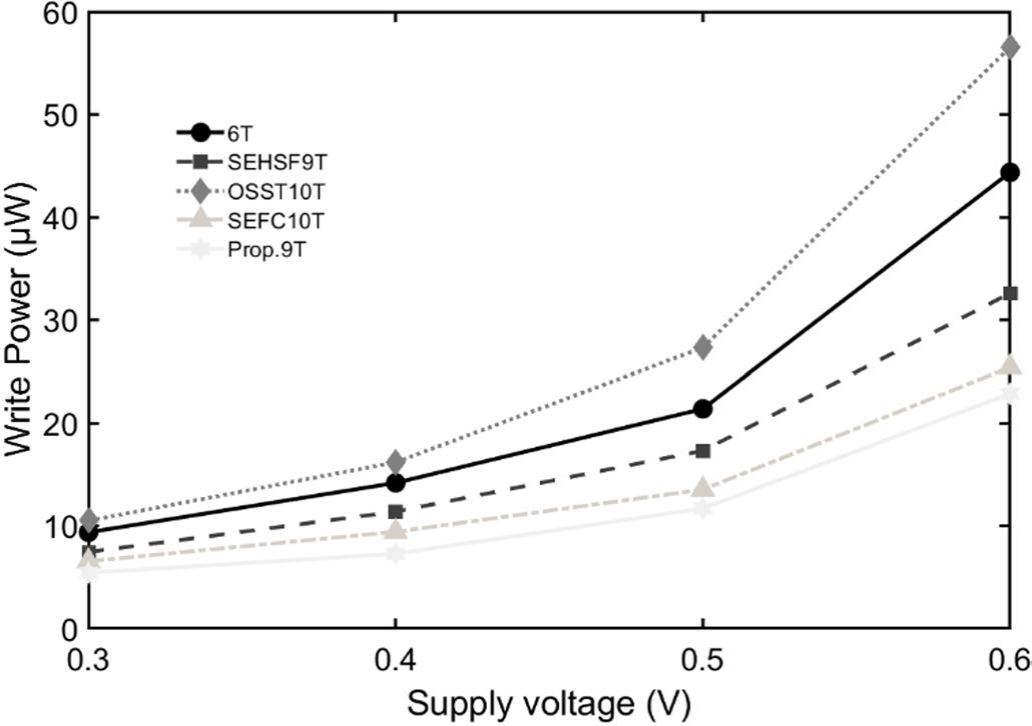
Write power values of the investigated SRAM designs at a range of supply voltage values (0.3 to 0.6 V).

The write power consumption of the tested SRAM designs across various supply voltages is presented in Fig. 16. The 6T and OSST10T designs, utilizing fully differential and pseudo-differential write structures respectively, exhibit higher write power compared to the SEHSF9T, SEFC10T, and the proposed designs. However, due to its higher number of wordlines, the OSST10T consumes more write power than the 6T.

Conversely, the proposed 9T SRAM design not only employs fewer column-based wordlines compared to the SEFC10T and SEHSF9T designs but also features a cut-off in the power-to-ground path of its inverters during a write operation. This mechanism contributes to a significant reduction in its write power consumption. At a 0.3 V supply, the proposed 9T design consumes 5.44 µW per write operation. This represents a 42.0% reduction compared to the 6T and a 48.2% reduction compared to the OSST10T. Furthermore, it achieves write power savings of 27.1% and 17.2% relative to the SEHSF9T and SEFC10T designs, respectively.

### Leakage power

The leakage power parameter as a static power generating component is considered one of the key indicators in the performance evaluation of a memory architecture in the data retention phase. The importance of this parameter comes from the fact that mainly SRAM cells work in a static state during their operation. On the other hand, in single-digit scale technologies, subthreshold leakage current accounts for a dominant share of static power^12,14,43^.

**Fig. 17:**
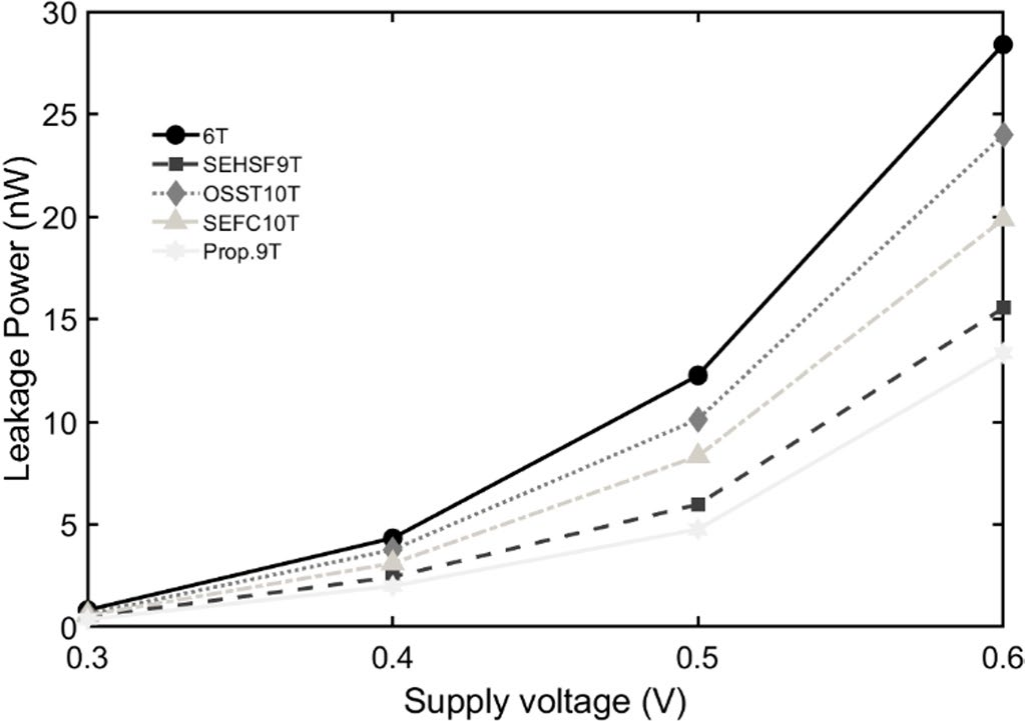
Leakage power values of the investigated SRAM designs at a range of supply voltage values (0.3 to 0.6 V).

According to the results presented in Fig. 17, the leakage power behavior in different SRAM designs changes as a direct function of the cell supply voltage. Among the investigated architectures, the common six-transistor buffer structure due to the use of enlarged transistors, especially in crossover inverters to solve the competition between the read and write paths, has the highest amount of leakage. Also, the structure of OSST10T shows a relatively high leakage power due to the additional leakage path in the inverse based on Schmitt-trigger through a feedback transistor.

Compared to these structures, SEHSF9T architecture has less leakage power compared to SEFC10T cell due to the elimination of RBL leakage, the use of stacking of transistors in the latch core and the reduction of the number of transistors. However, among other schemes, the lowest leakage power belongs to the proposed memory structure. The superior performance of the first cell is mainly due to the effective stacking of the transistors, which significantly reduces the subthreshold leakage current by increasing the voltage of the inner intermediate nodes. In addition, the floating RBL in the proposed memory architecture also plays an important role in reducing the leakage power.

At the cell supply voltage of 0.3 V, the proposed structure achieves a leakage power equal to 0.36 μW, which is the lowest value among all the compared architectures. Specifically, this value represents a 56.1% reduction and a 42.9% reduction compared to the OSST10T and conventional six-transistor architectures, respectively. In addition, the leakage power of the proposed structure is 25.0% and 35.7% lower than the counterpart architectures SEHSF9T and SEFC10T, respectively.

### DC stability performance statistical analysis

To evaluate the stability and reliability of other CNTFET SRAM architectures, extensive statistical analyses based on MC simulations have been performed. In this regard, 1000 random samples have been used to realistically incorporate the effects of process variations. Key process parameters including carbon nanotube diameter, oxide thickness, structural pitch, channel length, and nanotube density have been assumed to be based on independent Gaussian distributions. The variations of each of these parameters are within ± 15% around their nominal value, which corresponds to a deviation of ± 3σ^41^. Such an analysis is essential and important to understand the impact of manufacturing variability on SRAM cell performance, because even small deviations in these fundamental parameters can directly affect the stability and performance accuracy of the memory architecture.

**Fig. 18:**
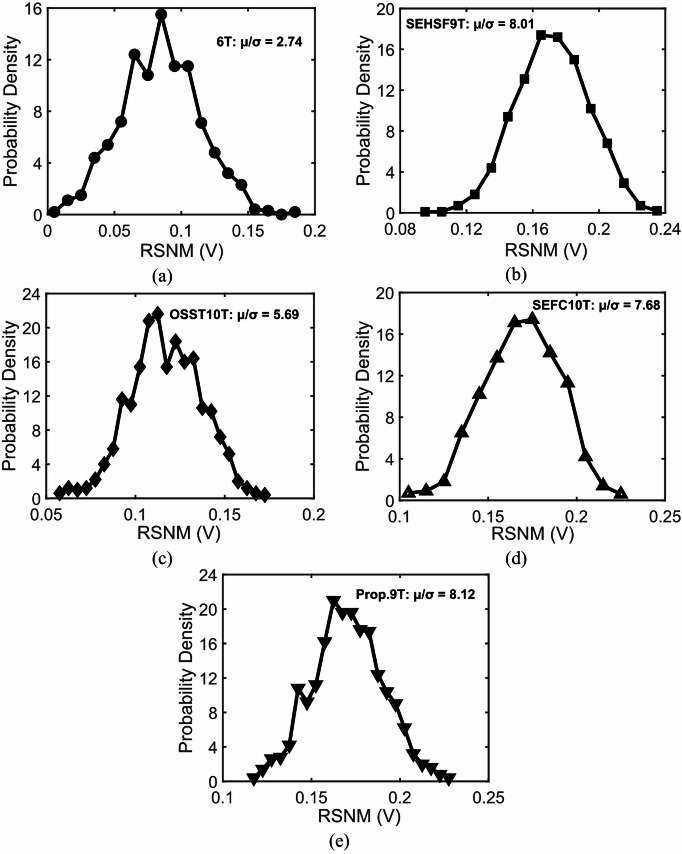
1000-run MC simulation-based RSNM distribution plots of the investigated SRAM designs at 0.3 V supply voltage. (**a**) 6T, (**b**) SEHSF9T, (**c**) OSST10T, (**d**) SEFC10T, and (**e**) proposed 9T.

**Fig. 19:**
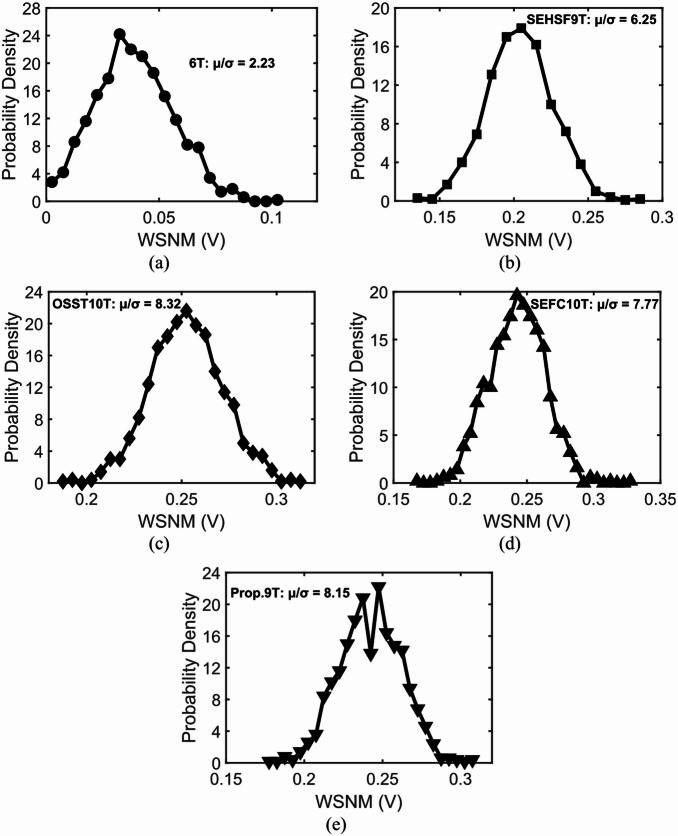
1000-run MC simulation-based WSNM distribution plots of the investigated SRAM designs at 0.3 V supply voltage. (**a**) 6T, (**b**) SEHSF9T, (**c**) OSST10T, (**d**) SEFC10T, and (**e**) proposed 9T.

Based on the results of the simulations of the statistical distribution of RSNM and WSNM at a cell supply voltage of 0.3 V in this section, it is possible to evaluate the behavior of the designs under real operating conditions. The graphs presented in Figs. 18 and 19 confirms the importance of process-based analysis in ensuring the reliability of memory circuits in emerging technologies such as CNTFET.

To quantitatively compare the resistance of other architectures to process changes, the ratio of the mean to the standard deviation (μ/σ) is considered as a reliability index. The results indicate that the proposed structure has the highest μ/σ value of 8.12 from the RSNM criterion, which indicates superior cell read stability compared to other counterpart architectures. In line with the evaluation of the WSNM parameter, the proposed nine-transistor structure has also shown very good performance and has achieved a μ/σ ratio of 8.15. Although this value is slightly lower than the OSST10T design, it significantly exceeds the 6T, SEHSF9T, and SEFC10T structures. These encouraging results indicate the high robustness of the proposed architecture in both read and write operation phases, establishing it as a reliable candidate for implementing SRAM based on the emerging CNTFET technology. The summary of μ/σ values for both RSNM and WSNM factors based on a cell supply voltage of 0.3 V is presented in Table 7.

**Table 7:** Mean to standard deviation ratios (*μ*/*σ*) for RSNM and WSNM of the investigated SRAM designs at 0.3 V supply voltage.

SRAM cell	*μ*/*σ* of RSNM	*μ*/*σ* of WSNM
6T^36^	2.74	2.23
SEHSF9T^28^	8.01	6.25
SEFC10T^27^	7.68	7.77
OSST10T^37^	5.69	8.32
Prop.9T	^8.12^	8.15

This study emphasizes the necessity of performing process variation analyses in realistic evaluation of SRAM architectures, since circuit performance in small-scale technologies is highly dependent on fabrication deviations. The superiority of the proposed cell in RSNM and WSNM indices shows that this architecture has the ability to effectively tolerate adverse effects caused by process variations and can be proposed as a stable and reliable memory solution in platforms with architectural level design based on CNTFETs technology.

### Area estimation

**Fig. 20:**
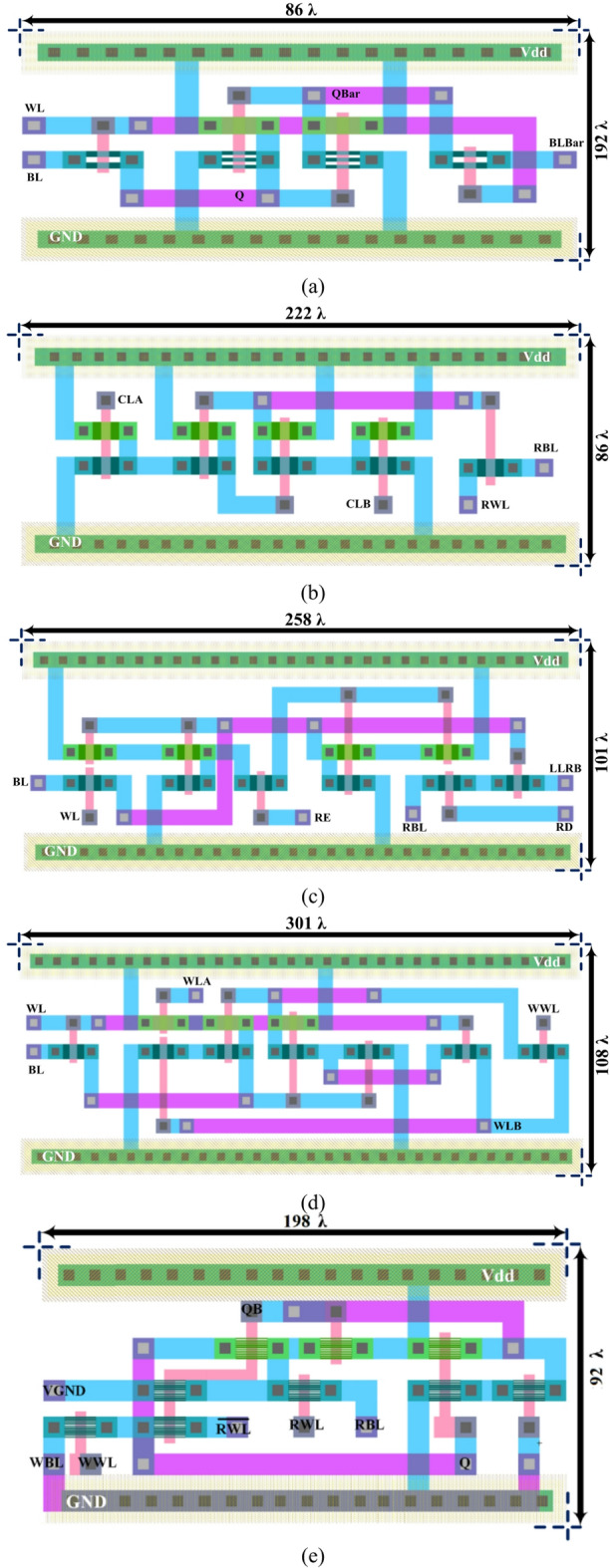
Layout schematics of the investigated SRAM cells. (**a**) 6T, (**b**) SEHSF9T, (**c**) SEFC10T, (**d**) OSST10T, and (**e**) proposed 9T.

Figure 20 illustrates the physical layout implementations of the proposed SRAM cell and the reference SRAM architectures, all realized using an identical CNTFET process framework as specified in Ref.^44^. The layouts were developed using the CAD Electric VLSI design environment with the *mocmos-cn* library^44,45^. During layout generation, careful attention was given to the required number of carbon nanotubes per transistor and to the pitch constraints compatible with 32-nm CNTFET technology^45,46^. To ensure an unbiased evaluation, the silicon footprint of each SRAM cell is reported in Table 8 using normalized λ-based units, where λ corresponds to 16 nm. The proposed 9T SRAM cell occupies an area of 18,216 *λ*^2^. When benchmarked against the conventional 6T SRAM cell, this represents an area overhead of 10.3%, which is an acceptable trade-off for the achieved improvements in stability and operational robustness. In contrast, relative to other state-of-the-art SRAM designs, the proposed cell exhibits a notable reduction in layout area, highlighting its superior area efficiency while maintaining enhanced reliability characteristics. Specifically, the proposed 9T SRAM achieves area reductions of 30.1%, 4.6%, and 44.0% when compared with the SEFC10T, SEHSF9T, and OSST10T designs, respectively.

**Table 8:** Area estimation of the studied SRAM cells based on their layout schemes.

SRAM cell	Layout area (${\lambda }^{2}$)	Area change versus 6T
6T^36^	16,512	Reference
SEHSF9T^28^	19,092	15.6% increase
SEFC10T^27^	26,058	57.8% increase
OSST10T^37^	32,508	96.9% increase
Prop.9T	18,216	10.3% increase

#### Write-half-select problem mitigation

A method to remove the half-select problem is discussed here. Figure 2 presented the suggested 9T SRAM cell, this design employs control signals that operate on a per-row basis: the WWL and a V_GND_. When a standard write is performed on one chosen bitcell, every other memory cell located in that identical row is also influenced by these signals. It can therefore be concluded that with this particular 9T configuration, writing occurs to all cells in a row simultaneously, a point detailed in source^47^. In situations where writing to a full row is not the objective, the issue of half-select disturbance can be addressed by implementing the energy-saving write-back circuitry described in reference^48^.

**Fig. 21:**
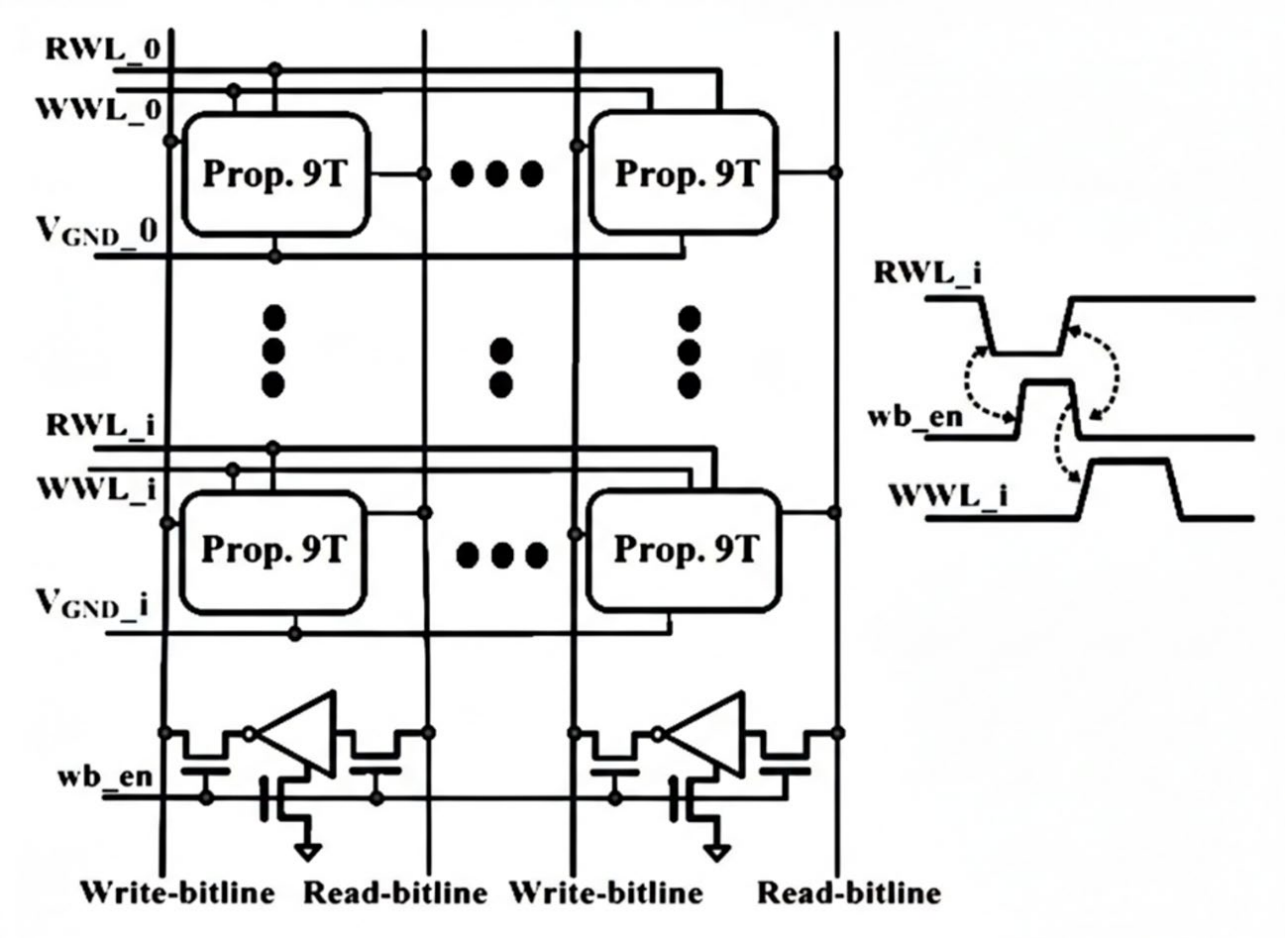
Write-back scheme used in the proposed 9T SRAM to mitigate the write-half-select disturbance problem.

The write-back process follows a sequence of steps. To begin, the read word line for the specific row in question is enabled. Activating this line allows the information held in the half-selected cells along that row to be detected via their dedicated read bitlines. This sensed data is subsequently transferred back to the matching write bitlines. A small circuit comprising three n-type transistors and an inverter manages this transfer, and it is governed by a separate write-back enable signal, illustrated in Fig. 21. Following this, the write-back enable signal is switched off. Deactivating it isolates the internal inverters from the read and write bitlines, preventing any disruption to the standard functioning of the memory array. Additionally, a transistor positioned at the lower end of these inverters is turned off to block any path for short-circuit or leakage current while the system is inactive. The final step is to activate the write word line for that row.

This approach resolves the half-select disturbance concern because during the write phase, the electrical charge present on the write bitlines does not adversely affect the internal voltages of the half-selected cells. The bitline voltages are maintained at levels equal to the internal node voltages of the cells, eliminating any damaging differential. Crucially, this method does not demand the complete and power-intensive sequence of a full read followed by a full write operation, as was suggested in reference^48^, leading to noticeably lower power consumption.

Two primary advantages provided by this internal write-back strategy contribute to enhanced performance for the SRAM. First, it entirely avoids the conventional read procedure, which would normally require enabling word lines, triggering sense amplifiers, setting latches, and other associated actions. Second, a complete formal write cycle is also unnecessary. The objective during write-back is merely to alter the voltage levels on the bitlines, a less demanding task that can be accomplished efficiently with just a compact inverter circuit^48^.

It is important to note that the proposed SRAM cell achieves half-select immunity in bit-interleaved arrays through a lightweight write-back scheme rather than requiring additional column-level assist circuitry. During a write operation, half-selected cells along the same row are temporarily read and their stored data is restored onto the corresponding write bitlines prior to activation of the write wordline. This approach avoids voltage disturbance at internal storage nodes without necessitating differential bitline control or extra column circuitry. Moreover, the write-back operation is implemented using a compact local circuit and does not involve a full read–modify–write cycle, thereby incurring minimal energy and delay overhead.

#### Soft-error mitigation

When high-energy particles hit sensitive areas in SRAM cells, they can cause temporary faults, known as soft errors, by creating charge. Smaller technology sizes mean these faults are more likely to spread to nearby areas, which increases the chance of multiple errors at once. So, SRAM cells are quite open to soft errors caused by radiation^49^.

The critical charge (*Q*_*C*_) measures how well an SRAM cell resists soft errors. It is the smallest charge needed to flip the stored data. The soft error rate (SER) relies heavily on *Q*_*C*_, following the formula in Eq. (5), where *N*_*flux*_ is the neutron flux density, *A* is the sensitive area, and *Q*_*S*_ is how well charge is collected. Because *Q*_*S*_ is usually quite small, even slight increases in *Q*_*C*_ can greatly lower the SER^49^.

5 $$SER = N_{flux} \cdot A \cdot e^{{ - Q_{S} /Q_{C} }}$$

To assess how well the SRAM cell resists radiation, a double-exponential current source simulates a particle hitting the storage node. Equation (6) shows the injected current, where ${\tau }_{r}$ and ${\tau }_{f}$ are the rise and fall time constants, representing swift ion-track creation and later charge collection, respectively^49^. Values of ${\tau }_{r}=50$ ps and ${\tau }_{f}=200$ ps are used, based on previous research^50^.

6 $$I_{inj} \left( t \right) = I_{peak} \left( {e^{{ - t/\tau_{f} }} - e^{{ - t/\tau_{r} }} } \right)$$

Nodes holding a logic ‘1’ are more prone to soft errors. Thus, the pulse in Eq. (6) is applied to the relevant storage node when the device is in standby. By changing the pulse strength, we find the smallest peak current (*I*_*peak*_) and critical time (*T*_*C*_) needed to flip the stored data. The *Q*_*C*_ is then found using Eq. (7). Larger *Q*_*C*_ values mean better protection against soft errors caused by radiation^7^.

7 $$Q_{C} = \mathop \smallint \limits_{0}^{{T_{C} }} I_{inj} \left( t \right) dt$$

**Fig. 22:**
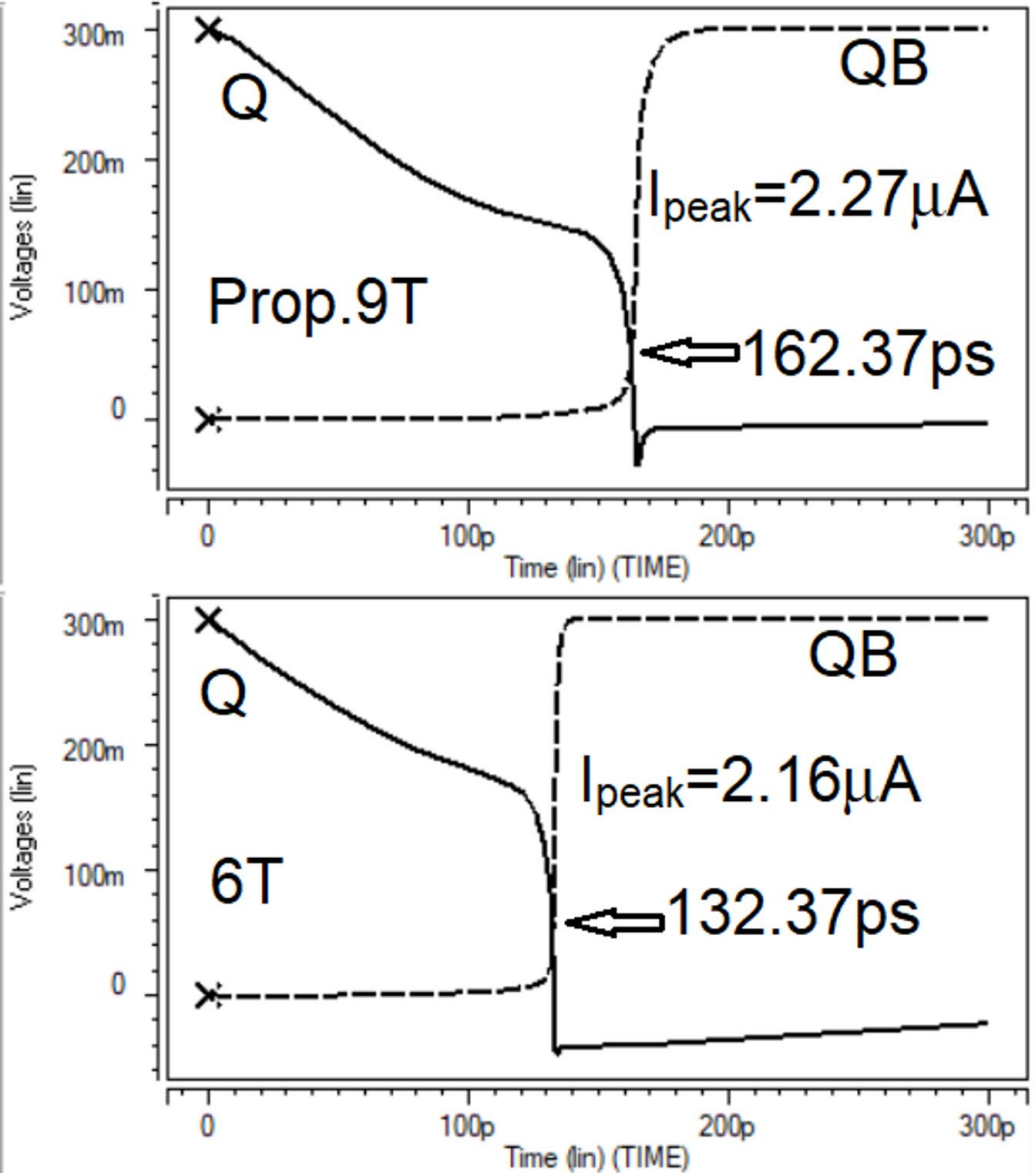
Voltages at nodes Q and QB for both 6T and proposed 9T SRAM cells to measure *T*_*C*_.

Through repeated simulations to pinpoint *I*_*peak*_, we found that a current of 2.16 µA is needed to flip the data in the 6T SRAM cell, whereas the proposed 9T SRAM cell needs 2.27 µA. Figure 22 illustrates the voltages at nodes Q and QB for both designs. The *T*_*C*_ for 6T SRAM cell is 132.37 ps, but it is 162.37 ps for the proposed 9T design. Based on Eq. (7), the proposed 9 T SRAM design shows a *Q*_*C*_ value of 0.144 fC, about 32% greater than the 6T SRAM design at 0.3 V.

It is noted that the critical charge is influenced by both particle energy and strike location. In this work, a worst-case scenario is considered by injecting charge at the most sensitive storage node using standard double-exponential parameters. While variations in particle energy and strike position may affect the absolute QC values, the relative comparison among different SRAM designs remains valid under identical simulation conditions.

## Conclusion

This work presented a robust and energy-efficient CNTFET-based 9T SRAM cell that effectively addresses the key challenges of stability degradation, power dissipation, and process variability in nanoscale memory design. By fully decoupling the read and write paths and employing a single-ended access scheme, the proposed architecture eliminates read-disturb and half-select failures while significantly enhancing noise margins at low supply voltages. At 0.3 V, the proposed cell achieves an RSNM equal to its hold SNM, a 2.1 × improvement in RSNM, and over a 14 × enhancement in WSNM compared to the conventional 6T SRAM. In addition, reduced bitline activity and effective transistor stacking enable the lowest read, write, and leakage power among the compared designs. Monte Carlo simulations further confirm strong robustness against process variations, demonstrating superior *μ*/*σ* ratios for both RSNM and WSNM. These improvements are realized with only a modest area overhead relative to the 6T cell and a smaller footprint than existing 9T and 10T alternatives, establishing the proposed 9T SRAM as a promising solution for low-voltage, energy-constrained CNTFET-based memory systems.

## Data Availability

All data generated or analyzed during this study are included in this published article.
